# The Use of Social Networking Sites and Pro-Environmental Behaviors: A Mediation and Moderation Model

**DOI:** 10.3390/ijerph18041805

**Published:** 2021-02-12

**Authors:** Zakir Shah, Lu Wei, Usman Ghani

**Affiliations:** 1 College of Media and International Culture, Zhejiang University, Hangzhou 310058, China; zakir@mail.ustc.edu.cn; 2 Department of Business Administration, Iqra University, Karachi 75500, Pakistan; gusman@mail.ustc.edu.cn; 3 College of Education, Zhejiang University, Hangzhou 310058, China

**Keywords:** attention deficit, climate change, decision-making self-efficacy, fear of victimization, pro-environmental behaviors, social networking sites

## Abstract

Climate change poses a huge threat. Social networking sites (SNSs) have become sources of human–environment interactions and shaped the societal perception of climate change and its effect on society. This study, based on the extended parallel process model, aims to examine the effect of exposure to climate change-related information on SNSs on the pro-environmental behaviors of individuals. The study examines the mediation effect of fear of victimization from climate change between the exposure to climate change-related information on SNSs and pro-environmental behaviors, including the moderation effect of attention deficit and decision-making self-efficacy with the help of appropriate instruments. A total sample of 406 reliable questionnaires were collected from students using SNSs in China, and data were analyzed through SPSS and AMOS. Results indicate that the exposure to climate change-related information on SNSs has a direct positive effect on users’ pro-environmental behaviors (β = 0.299, *p* < 0.01). Fear of victimization from climate change also mediates the relationship between exposure to climate change-related information on SNSs and pro-environmental behaviors (β = 0.149, SE = 0.029, *p* < 0.01). In addition, attention deficit moderates the relationship of exposure to climate change-related information on SNSs with fear of victimization from climate change (β = −0.090, *p* ≤ 0.01) and pro-environmental behaviors (β = −0.090, *p* ≤ 0.05). Similarly, the relationship between fear of victimization from climate change and pro-environmental behaviors is moderated by decision-making self-efficacy (β = 0.267, *p* ≤ 0.01). The findings offer implications for media organizations and government policy makers, who should post or spread environmental information through the most trustworthy media, with trustworthy sources, in an effective manner, and without exaggerated adverse impacts.

## 1. Introduction

The frequency of problems such as water scarcity, deforestation, and greenhouse gas emissions has risen as a result of climate change [1]. The Intergovernmental Panel on Climate Change describes climate change as “any climate change over time, whether due to natural variability or as a consequence of human activity” [2]. Similarly, climate change is directly or indirectly due to human actions that change the nature of the global environment and take place over a longer period of time, normally decades or longer [3]. 

Nevertheless, media (mass media and social networking sites (SNSs)) play a critical role in providing environmental education, increasing people’s knowledge, and shaping the societal perception of climate change and its effect on society [2,3,4,5,6]. SNSs reach a large number of people, attract user attention toward environmental problems, raise awareness, and provide information about the adverse impacts of climate change on civil society [7,8]. SNSs can significantly influence individual and collective ecological concerns, environmental attitudes, and users’ pro-environmental behaviors [9,10]. Pro-environmental behaviors refer to the purposeful actions at the individual or community level that benefit the environment or at least decrease the negative effects on the environment [11,12,13]. 

Previous research revealed that threatening messages on media that contain severity and susceptibility information generate fear, evoke anxiety, and influence the protective motivations of individuals [14]. Similarly, fear of climate change refers to the sacredness of individuals to the serious effects of climate change [15,16]. The perceptions of severity and vulnerability encouraged by fear-appeal messages may function as core relational themes of fear and anxiety [17]. Research reveals that the recipients of fear appeal may act to mitigate the perceived threat of climate change [16]. Communication practitioners use emotions in news stories, advertisements, and in other programs on media to attract people’s attention and influence their attitudes and behaviors [18]. Recent research shows a strong combination of persuasive messages, mass media exposure levels (TV, news), and public concerns about various issues [19]. Specifically, fear-inducing messages are primarily used in health communication persuasion and prevention campaigns. Nevertheless, the role of SNSs has recently emerged as a significant concept in inducing successful and continuous changes in behavior for users regarding climate change and its impact on society, which needs further exploration. To fill this research gap, the study attempts to explore and identify how exposure to climate change-related information via SNSs elicits fear of victimization in users that in turn may influence their pro-environmental behaviors. The study investigates this effect through a quantitative survey approach. 

Whether people are willing to tackle the perceived danger of climate change or contribute to it is a concern [20], because despite their fear of climate change, motivating individuals to exhibit pro-environmental behaviors is difficult [21]. Individuals must have strong and high-level decision-making self-efficacy to engage in mitigation actions. Self-efficacy refers to people’s beliefs in their capabilities and potential to perform or deal with a given behavior or task [22]. This study replicates self-efficacy with decision-making self-efficacy, suggesting that people with high-level and strong decision-making self-efficacy set difficult goals and exert additional effort toward pro-environmental behaviors. The study further examines the moderating effect of decision-making self-efficacy on the association between fear of victimization and pro-environmental behaviors. 

Previous research showed that access to additional information on media is the best motivator of the pro-environmental behaviors of people [23,24]. However, with the expanding size of SNSs, users encounter an endless flow of information with easy access that is beyond their cognitive abilities to process [25]. Due to attention deficit, people face difficulties in managing, properly focusing, and filtering the quality of available information [26]. The attention of people to the media coverage and portrayals of various issues influence their perceptions and attitudes [27]. However, with limited attention, the expanding number of information feeds may negatively affect the intentional behaviors and decision-making capabilities of individuals [28]. This study describes attention as the tendency to direct cognitive resources on climate change-related information on SNSs. Previous research indicates that paying attention to information allows people to engage in the effective communication and turn the information to which they have been exposed into their personal awareness, leading them to pro-environmental behaviors [29,30]. Thus, this study also examines how the attention deficit buffers the association of exposure to climate change-related information on SNSs with the emotional reactions and pro-environmental behaviors of users. 

It also contributes to the theoretical knowledge on the effect of media coverage about environmental issues on the pro-environmental behaviors of people. A comprehensive theoretical model, which demonstrates that exposure to climate change-related information on SNSs significantly induces fear of victimization and positively influences the pro-environmental behaviors of individuals, is presented. Their attention toward climate change-related information on SNSs moderates these relationships. In addition, the study demonstrates that fear of victimization from climate change mediates the association between exposure to climate change-related information on SNSs and the pro-environmental behaviors of individuals. Their high decision-making self-efficacy may strengthen the association between fear of victimization from climate change and pro-environmental behaviors. 

The study is divided into different sections; first, the research background and introduction are presented. Second, the theoretical framework and development of hypotheses are explained. Third, the methodology and findings of the study are discussed. Fourth, the findings, theoretical and practical implications, limitations, and future directions are presented. Last, a short conclusion is provided.

### Study Area

China is situated geographically in the middle and low latitudes, covering an area of approximately 9.6 million km^2^ [31]. Previous research shows that China is significantly affected by climate change due to its geographical nature [32], and its temperature may rise in the future [33]. Similarly, with 1.37 billion people, China is the country with the highest population [34], and has the world’s second largest economy [1]. The high demand for food production, rapid development, accelerated urbanization, and agricultural land changes have made China’s economy potentially vulnerable to climate change [35]. In addition, research has shown that climate change has adversely affected agricultural production, which could pose a risk to food security [36]. Moreover, agricultural development that is dependent on the natural climate is also more vulnerable to drought and flooding. For example, as a result of climate change, in certain regions crop land is facing a water scarcity crisis, while in some other areas there is a threat of flooding which has a negative effect on the growth of the agrarian economy in China [37]. Past research has shown that climate change can lead to more frequent and serious floods, which will increase health problems in vulnerable populations, reduce the quality of human life, and impede economic development [38]. In 2016 in Anhui province, for instance, a devastating flood impacted 12.82 million people causing direct economic losses of USD 8.25 billion, while the risk of infectious diarrhea in exposed populations increased significantly [39]. In 2003, the entire basin was adversely affected by the Huaihe River flood and the economic losses of the provinces of Jiangsu and Anhui reached 33 billion Yuan [37]. More recently, about 420,000 people in the province of Anhui were affected by heavy rain in 2020. Based on the above details, it is essential to investigate the mechanism behind the pro-environmental behaviors of individuals in China.

## 2. Theoretical Background

The appraisal theory of emotion suggests that emotions are dynamic processes and are elicited with the observations and cognitive illustrations of environmental events [40]. Emotions arise as a result of the appraisal processes of the environments with which they have relationships in a certain way [14]. Drawing from appraisal theory, the extended parallel process model (EPPM) describes that an individual’s perception of a threat and perception of efficacy jointly regulate the response behavior of a person to an issue [41,42]. Research reveals that EPPM is one of the most suitable models for assessing the behaviors of people [43]. It examines how individuals are informed and how that information or knowledge influences their motivations to take action [44]. EPPM is based on two basic concepts, namely, threat concepts (perceived severity and perceived susceptibility) and efficacy concepts (response efficacy and self-efficacy) [45,46]. EPPM describes that the cognitive appraisal of threats arises from the environmental stimuli according to the severity of threats and the susceptibility of individuals to such threats. Perceived severity refers to the perception of a person about how serious the outcome of a disaster may be if he/she becomes the victim of it, whereas perceived susceptibility describes how likely he/she may be affected. Perceived susceptibility indicates the perception of a person that the threat of a disaster or crisis may affect him/her personally [41]. Individuals also evaluate their self-confidence in dealing with the perceived threat of a disaster [47]. Similarly, response efficacy refers to the effectivity of a proposed or recommended response for avoiding or mitigating the threat of a disaster. Individuals with high efficacy attempt to react and formulate the nature of their responses to the perceived threat of a disaster [48,49]. The perceived threat and confidence level of a person determine his/her motivations and actions.

EPPM explains how individuals adapt disaster-related information that is deemed fearful or threatening [47]. EPPM theory predicts that exposure to disaster-related information through different sources generates fear in individuals that in turn influences their cognitive and emotional responses to the perceived threats [50]. According to Witte [51], fear-inducing experiences are effective in changing individuals’ adaptive and maladaptive behaviors through their exposure to disaster-related information. Similarly, individuals appraise disaster-related information by threat or efficacy, which results in three reactions, namely, indifference, information rejection, and information acceptance [43]. However, Witte [51] found that repeated exposure to threat-related information/messages does not change the actual or perceived threats to individuals but may change the efficacy of responses. In the case of high perceived threat and high perceived efficacy, individuals are predicted to cognitively manage the threat and employ the proposed responses to prevent it, known as the danger control process. Further research indicates that when the perceived threat of a disaster is higher than the perceived efficacy of individuals, the fear control process dominates, in which individuals emotionally deal with the perceived threat instead of performing the recommended responses [17]. Similarly, if the perceived efficacy is lower than the perceived threat, then individuals become afraid and reduce their fear by rejecting their perception of the threat. When the perceived efficacy outweighs the perceived threat or the repeated exposure to disaster information reduces the perceived threat, the danger control process motivates individuals to adaptively deal with the perceived threat of a disaster [47]. Individuals with high efficacy attempt to react and formulate the nature of their responses to the perceived threat of a disaster [48,49].

EPPM theory is one of the several psychological theories that have been recommended for demonstrating the disaster reactive behaviors of individuals. Researchers know little about the effects of SNSs on the fear arousal and pro-environmental behaviors of persuasion users. Most previous studies on health communication applied EPPM, whereas recent studies suggest EPPM as an explanatory model for risk communication, which must be explored thoroughly. Individuals decide to engage in pro-environmental behaviors on the basis of their perceived risk assessment (perceived severity and adverse consequences of an event) and coping appraisal (response and self-efficacy) [52,53,54,55]. Prior research found that the use of efficacy-based messages in combination with the threat components promoted waste separation behaviors of female students in Iran [56]. Similarly, Xue et al. [57] found a significant effect of EPPM based messages on public engagement with climate change (intention to seek more information about climate change and take protective actions). Therefore, investigating how the exposure of people to climate change-related information on SNSs induces fear of victimization from climate change and how it influences their pro-environmental behaviors is necessary and useful. Drawing on EPPM, this study examines the effect of exposure to climate change-related information on SNSs on the fear arousal and pro-environmental behaviors of users.

### 2.1. Exposure to SNSs

Previous research identified the effect of media exposure on the emotional reactions of people. Among the numerous outcomes of media effects, the contribution of the present study concerns the exposure to climate change-related information on SNSs, fear of victimization, and pro-environmental actions of people. Media exposure assessments are important for media impact research in communication science [58,59]. Scholars describe media exposure as “the degree to which people receive messages or media materials, whether or not they are adequately heard to be recognized” [60]. In this study, exposure to climate change-related information describes the quantity of information heard or viewed on SNSs concerning the climate change. Such exposure to climate change-related information can enhance individuals’ understanding of environmental issues which ultimately leads them to engage in pro-environmental behaviors [30]. Previous studies revealed a strong relationship between exposure to SNSs and people’s fearful reactions to various issues [61,62,63,64]. Graphical warning notices and information on disaster-related media may also trigger people’s fear [18,65].

High exposure to climate change-related information on media has positive relationships with the environmental concerns and environmental knowledge of people [66,67]. The framing or the way of presenting messages influences audience responses and problem views and promotes the importance of information among people [68]. People experience different emotions, such as hope, worry, fear, anxiety, and humor, while exposed to information about climate change [15,69,70]. Fear arises when an individual perceives a personal physical threat and is supposed to be a useful motivational tool toward his/her pro-environmental behaviors [71]. Negative emotions (e.g., fear) about perceived risks and threats generate great persuasion and induce careful processing about the alleviation of the perceived risks of climate change [71]. Therefore, the study suggests the following:

**Hypothesis** **1.** *Exposure to climate change-related information on SNSs is positively related to the fear of victimization from climate change in users.*

**Hypothesis** **2.** *Exposure to climate change-related information on SNSs is positively related to the pro-environmental behaviors of users.*

### 2.2. Mediation Effect of Fear of Victimization from Climate Change

According to fear appeal, stakeholders and communicators use the dominant communication approach to raise awareness regarding environmental issues, instill fear, and motivate people’s behavioral intentions toward climate change mitigation [21,72]. Information on fear appeal has two forms. The first type is linked to the threat and its importance to the receiver of the information, whereas the second type focuses on the reaction of the recipient to the threat [73]. Fear is a multifaceted reaction that includes negative emotional responses to a real or perceived threat [74,75].

The prospect of the catastrophic effects of climate change, particularly with regard to the occurrences of crises such as floods, drought, heat waves, tempests, and emissions, may terrify people, affect their opinion, perceptions, behaviors, and decision making to avoid or mitigate the negative impacts of climate change [23,76]. According to Witte [51], people whose efficacy is higher than their risk perception fall in an adaptive state of danger control to manage their risk, whereas people whose efficacy is lower than their risk perception fall in their maladaptive state of fear control to manage their fear. People with fear of climate change regulation likely deny climate change information by avoiding or reacting to such information. People in hazardous control tend to engage further in various approaches for resolving the danger and in environmental activism [77]. Scholars use fear in the kind of imagery in association with climate change and find it an effective tool for people’s motivation and engagement in pro-environmental behaviors [15,21]. Past research revealed that fear appeal messages can significantly influence the defensive behaviors of individuals to avoid or mitigate the negative outcomes of disasters [78]. Fear appeal indicates messages that are designed to scare the target audience by describing a serious threat to them. The purpose of fear appeal message is to motivate and engage the intended audiences in certain behaviors based on a fear [78]. Media increase the personal relevance of environmental risk information and induce fear and anxiety from the environmental hazards [4]. The pro-environmental behaviors of individuals have a major influence on the fear of exposure to climate change-related information. The evaluation process of an individual assesses whether or not the stimulus is in his or her favor causing fear, which, in turn, leads to behavioral reactions (e.g., searching for punishment or treatment) [79]. In this study, climate change-related information includes content illustrating the adverse effects of climate change. Such information can be linked to pro-environmental behaviors as individuals learn about the severity of climate change and its vulnerability. In addition, being exposed to threat information will evoke the fear of individuals and that fear increases their perception of severity and vulnerability, eventually influencing their actions to take some kind of prevention measures [80,81,82]. Therefore, this study hypothesizes that:

**Hypothesis** **3.** *Fear of victimization from climate change is positively related to the pro-environmental behaviors of users.*

**Hypothesis** **4.** *Fear of victimization from climate change mediates the association between exposure to climate change-related information on SNSs and the pro-environmental behaviors of users.*

### 2.3. Moderation Effect of Attention Deficit and Decision-Making Self-Efficacy 

People are exposed to considerable information on SNSs that competes to gain attention for cognitive process [83]. However, an endless flow of information on SNSs with easy access is beyond the cognitive abilities of users to process all these information [25]. An overwhelming amount of information on mainstream and Internet-based media competes for the cognitive resources of individuals [84] and is associated with attention deficit in individuals [85]. Attention is a bottleneck for information flow in the cognitive system of individuals that enables their selective mental processing. Attention directs individuals to prefer some information over the rest and links their perception, cognition, and action [86]. 

Individuals have limited cognitive resources by which they cannot process all aspects of environment at the same time. Nevertheless, attention processes the aspects that are considered most relevant to the interest and well-being of individuals and facilitates their adaptive behaviors [87]. According to Nicks and Carriou [88], emotional stimuli automatically create the attention engagement of individuals and then enhance it on the basis of their personal goals and motivations. Similarly, individuals’ emotional stimuli have a unique relationship with their attention [87]. That is, the amount of attention paid to information on media is significantly associated with the cognitive and emotional responses of individuals [89]. 

Past research indicated that media attention is an important prediction of information influence and is essential for persuasive effects [90]. Similarly, individuals’ attention toward information leads to their cognitive elaboration and learning from media. People’s attention to media information depends on its salience, interest, and relevancy [91]. Therefore, this study predicted that the attention of individuals can be an important determinant of their emotional and behavioral responses to climate change-related information on SNSs. Climate change-related information includes media content that aims to inform the public about pro-environmental behavior and climate change mitigation. When individuals pay attention to information, they become knowledgeable about the climate change-related issue. Individuals with high attention toward climate change-related information on SNSs likely elaborate on and acquire knowledge from the information [92]. Similarly, the more attention individuals pay to climate change-related information, the more the effect of media increases on their perceptions and behaviors. Likewise, the information deficit model described the lack of knowledge as a barrier to behavioral change [80]. The current study, therefore indicates that the attention of people to climate change-related information on SNSs can have a similar influence on their fear of victimization from climate change and pro-environmental behaviors.

Therefore, the study assumes that:

**Hypothesis** **5.** *The attention deficit of individuals to climate change-related information on SNSs moderates the association between exposure to climate change-related information on SNSs and their fear of victimization. When the attention deficit is high, the relationship between exposure to climate change-related information on SNSs and fear of victimization from climate change becomes weak and vice versa.*

**Hypothesis** **6.** *The attention deficit of individuals regarding climate change-related information on SNSs moderates the association between exposure to climate change-related information on SNSs and pro-environmental behaviors. When this attention deficit is high, the relationship between exposure to climate change-related information on SNSs and pro-environmental behaviors becomes weak and vice versa.*

### 2.4. Decision-Making Self-Efficacy

Self-efficacy plays a crucial role in influencing one’s choice behavior, efforts to overcome or mitigate obstacles, feelings of stress and anxiety, performance, and coping behaviors [93]. High self-efficacy also plays a critical role in gaining motivation, making decisions, and taking actions, whereas low self-efficacy is associated with negative thoughts, performance, and personal developments, all of which lead individuals into depression and anxiety [94]. Self-effectiveness refers to the trust in the ability of an individual to perform a particular task and produce desired results [22] and is a crucial factor of behavior change [95]. Previous studies showed a positive association between an individual’s self-efficacy and behavior change in different areas, such as decision-making self-efficacy and career indecision [96], travel behavior change [97,98], weight control and exercise behaviors [95], media use and pro-environmental behaviors [99], and self-efficacy beliefs and environmental literacy [100]. Furthermore, self-efficacy functions as a mediator [101], which encourages pro-environmental behaviors, such as recycling behaviors [102], reducing energy consumption, and using reusable shopping bags [103]. However, negative emotions significantly interfere with an individual’s thoughts and disturb the attentional resources that may negatively affect the decision-making process [104]. An individual with inferential efficacy beliefs utilizes and processes the available information that is associated with a rational and comprehensive decision making and meets the best interests of the individual [105]. 

In this study, decision-making self-efficacy is conceptualized as a cognitive assessment of the performance competencies of individuals to activate motivation, cognitive resources, and behaviors required to cope with climate change [103,106]. Previous research revealed that individuals with low decision-making self-efficacy likely decrease or completely give up their efforts in difficult situations [93]. Therefore, this study suggests that users’ decision-making self-efficacy is an important factor that may enhance the relationship between their fear of victimization from climate change and pro-environmental behaviors. People with high decision-making self-efficacy have more capacity to deal with difficult situations and be less likely experience negative emotions, whereas individuals with low decision-making self-efficacy more likely decrease or completely give up their efforts in difficult situations [85]. Thus, it assumes that:

**Hypothesis** **7.** *The decision-making self-efficacy of people moderates the association between their fear of victimization from climate change and pro-environmental behaviors. When their attention is high, the relationship between fear of victimization from climate change and pro-environmental behaviors becomes strong and vice versa.*

## 3. Materials and Methods

### 3.1. Participants and Process 

Data for the current study were collected from a large university in China during the Fall Semester, 2019, to test the proposed model of the study. The original version of questionnaire was in English that was translated into Chinese version. After the refinement of the final version, an online questionnaire was employed to collect the data. A link of questionnaire was sent to different groups and personal contacts in WeChat, and a statement was attached promising the confidentiality of participants. As the data were collected from university students, for this purpose, the study selected academics group at different level. Group administrators (normally instructors/professors) were contacted to help in the data collection. A total of 500 respondents were recruited, and responses were obtained from 445 (89%). Out of 445, 39 responses were eliminated due to incomplete responses. Finally, 406 responses were considered for further analysis. There were 53.4% male and 46.6% female participants in the study. Demographic details of the respondents are mentioned in Table 1 below.

### 3.2. Measurements 

The related frameworks and questionnaire, which were used to test the proposed research model, were built on the basis of previous studies. By using a five-point Likert scale, all items were measured.

#### 3.2.1. Exposure to SNSs 

The research adapted a three-item scale from Lee [66] to measure the exposure of individuals to climate change-related information on SNSs. In this research, the Cronbach’s alpha value was (0.939). 

#### 3.2.2. Attention 

To measure the attention levels of SNS users, the study adapted the eight-item scale used by Brooks [107], Chaffee and Schleuder [108], Namkoong et al. [89], and Rose et al. [27]. Participants were asked to estimate the degree to which each element represents their attention level to climate change-related information on SNSs. In this analysis, the Cronbach’s alpha of the scale was (0.973). 

#### 3.2.3. Fear of Victimization from Climate Change

A four-item scale was adapted from Williams and Akers [109] and Reyes-Sosa and Molina-Coloma [110] to measure the fear of victimization from climate change of SNS users. This scale was also utilized by Shah et al. [111]. In the present study, the Cronbach’s alpha value was (0.867). 

#### 3.2.4. Pro-Environmental Behaviors

To examine users’ pro-environmental behaviors, the eight-item scale used by Lee [66] was adapted. Its Cronbach’s alpha value was (0.964). 

#### 3.2.5. Decision-Making Self-Efficacy

To measure users’ decision-making self-efficacy, the six-item scale used by Myburgh et al. [104] and Nolan et al. [112] was adapted. Its Cronbach’s alpha was (0.955).

#### 3.2.6. Control Variables

A large number of previous research has examined various individuals’ factors such as gender, age, and educational level to the model to control for the possible impact of these factors on people’s pro- environmental behaviors [9,113,114]. Therefore, this study adds gender, age and education as control variables. Table 1 shows a list of control variables. 

### 3.3. Analytical Strategy

Data analysis were carried out through SPSS 22.0 and AMOS 21.0. Cronbach’s alpha, composite reliability, average variance extracted, and model fitness indices (CMIN/DF, SRMR, REMSA, CFI). were calculated to examine the constructs reliability and validity, and model fitness. The study utilized Hayes [115] PROCESS macro to estimate both mediation and moderation effects. In this study, a bootstrap analysis was conducted by using path analytic procedures to test the study hypotheses, and to assess the significance of the indirect effects [116,117,118]. Bootstrap is considered a more sophisticated and reliable method for estimating indirect effects in the social sciences [115,119]. These techniques were used in the recent studies [120,121]. 

## 4. Results

### 4.1. Reliability and Validity

Before checking the reliability and validity, the current research examined non-response bias issue by comparing early (n = 30) and late (n = 30) responses [122]. The t-test indicate no significant difference between these two groups (*p* > 0.05), indicating non-availability of non-response bias.

Constructs reliability and validity were assessed through Cronbach’s alpha (CA), factor loadings (FL), composite reliability (CR), and average variance extracted (AVE). Table 2 show the results of CA, FL, CR, and AVE, which indicates that all the values of CA, FL, CR, and AVE are in acceptable range recommended by Fornell and Larcker [123], Wu and Chen [124] and Hair, Gabriel, and Patel [125]. Moreover, Table 3 exhibits that all values of the square root of AVEs are greater than the variables inter-correlations, thereby affirming discriminant validity. Furthermore, CFA analysis was carried out to check the model fitness indices of the measurement model using AMOS-21. The resulting values fall within the widely agreed spectrum. RMSEA is *0.060*, which is less than the threshold value of *0.10* [126]. CMIN/DF is *2.443*, which fall within the recognized range as well. In addition, the CFI is *0.982*, and the SRMR is *0.047*; these values are both above the *0.90* estimates indicated [127]. The results show a good model fit of measurement. 

### 4.2. Correlations, Means and Standard Deviations

Table 3 describes the descriptive statistics and the means and standard deviations (SD) of the current study. The study also examined the correlations of control variables such as gender, age and education with the study variables and found no significant association with the main constructs of the study. In addition, ANOVA was used to examine the significant differences of different control variables. The findings showed that the pro-environmental behaviors did not display any significant differences among these control variables. Thus, the study did not include control variables for further analysis. The study findings were basically similar to or without the control variables; thus, the results are reported here without control variables [128].

### 4.3. Mediation Effect 

In this study, PROCESS macro suggested by Hayes [89] in SPSS was used to find a direct and indirect effect and test H1, H2, H3, and H4. The findings (See Table 4) indicate that the association between exposure to climate change-related information on SNSs and fear of victimization from climate change is positive and significant (β = 0.299, *p* < 0.01), which supported H1. The association between exposure to climate change-related information on SNSs and pro-environmental behaviors (total effect) is positive and significant (β = 0.149, *p* < 0.01), which supported H2. Similarly, the relationships between fear of victimization from climate change-related information on SNSs and pro-environmental behaviors is also positive and significant (β = 0.301, *p* < 0.01), leading to the acceptance of H3. Further, the findings show that the indirect coefficient was statistically significant because the confidence interval did not include zero (.094-.202), and also β = 0.090, SE = 0.016, and *p* < 0.01, which support H4. Thus, fear of victimization mediates the association between exposure to climate change-related information on SNSs and pro-environmental behaviors.

### 4.4. Moderating Effect of Attention Deficit and Decision-Making Self-Efficacy

**Figure 1:**
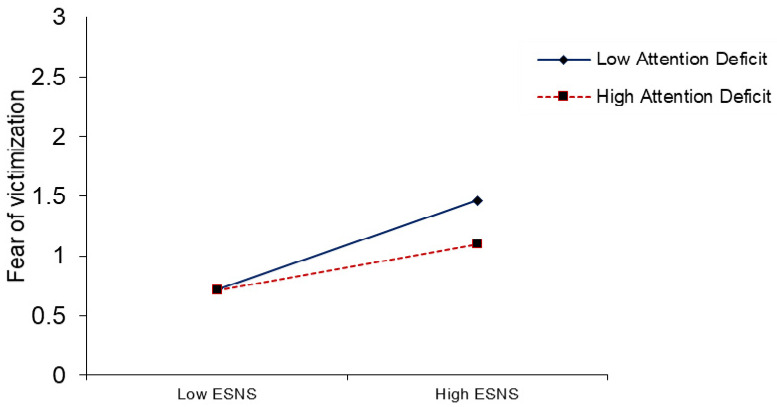
The moderating effect of an attention deficit on the relationship between ESNS and fear of victimization.

**Figure 2:**
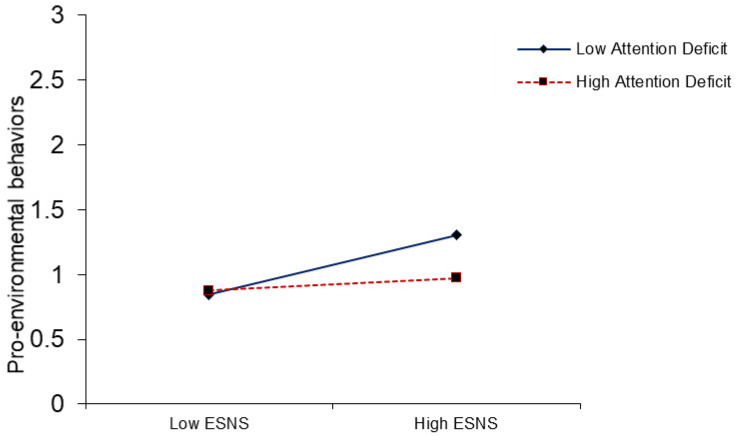
The moderating effect of an attention deficit on the relationship between ESNS and pro-environmental behaviors.

**Figure 3:**
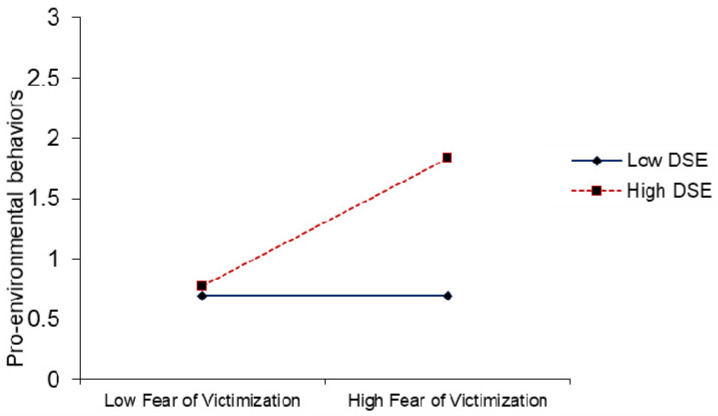
The moderating effect of decision-making self-efficacy on the relationship between fear of victimization from climate change and pro-environmental behaviors.

PROCESS macro was used to find the moderation effect suggested by Hayes [89]. The results of interaction term (ESNSs × attention) in Table 5, predicted that attention deficit moderate the association between exposure to climate change-related information on SNSs and users’ fear of victimization from climate change (β= −0.090, *p* ≤ 0.01). The positive relationship between exposure to climate change-related information on SNSs and users’ fear of victimization from climate change is weaker (β = 0.192, t = 2.715,) at high levels of attention deficit. The same relationship is positive and stronger (β = 0.372 **, t = 11.764,) at low levels of attention deficit (see Figure 1). Thus, H5 was supported. Similarly, in interaction term (ESNSs × attention), attention deficit moderates the direct relationships between exposure to climate change-related information on SNSs and pro-environmental behaviors (β = −0.090, *p* ≤ 0.05), leading to support the H6, as shown in Table 5. The positive relationship between exposure to climate change-related information on SNSs and pro-environmental behaviors is weaker (β = 0.049, t = 0.693,) at high levels of attention deficit. The same relationship is positive and stronger (β = 0.229 **, t = 7.242) at low levels of attention deficit (see Figure 2). Likewise, the interaction term (fear of victimization × DSE), the results show that an individual’s decision-making self-efficacy had a positive and significant moderating effect on the relationships between fear of victimization from climate change and pro-environmental behaviors (β= 0.267, *p* ≤ 0.01), supporting H7. The positive relationship between fear of victimization and pro-environmental behaviors is weaker (β = −0.002, t = −0.063) at low levels of decision-making self-efficacy. The same relationship is positive and stronger (β = 0.532 **, t = 7.524) at high levels of decision-making self-efficacy (see Figure 3).

## 5. Discussion

SNSs have become easy sources to be used in raising awareness by sharing different posts and information about climate change. Despite the growing number of studies on the role of media in raising social awareness regarding climate change, little has been identified about the socio-emotional reactions of people exposed to climate change-related information on SNSs. The findings revealed that exposure to climate change-related information on SNSs had significant and positive interactions with fear of victimization from climate change. The findings are in line with the results obtained by Cvetković, Öcal, and Ivanov [129] and Nellis and Savage [130] who found that exposure to media and wide-ranging media reporting of catastrophe produces diverse outcomes of risk perception and fear of victimization from disasters in individuals.

The findings also revealed that exposure to climate change-related information on SNSs had positive and significant effects on the pro-environmental behaviors of users. Evidence for supporting such findings comes from Huang [99], who found that exposure to climate change-related information on media has positive and significant effects on people’s pro-environmental behaviors. similarly, Liu and Han [131] indicated that Internet use directly and indirectly influence users’ pro-environmental behaviors. 

Regarding H3, the results indicated that fear induced by exposure to climate change-related information had significant positive relationships with users’ pro-environmental behaviors. The findings are in line with the results obtained by Kundzewicz, Matczak, Otto, and Otto [23], Hunter and Roos [15], and Chen [78] who revealed that individuals’ fear and uncertainty from climate change can influence their pro-environmental behaviors. Threatening climate change-related information induces fear in audiences and thus enhances its persuasive effect and pro-environmental behaviors [71]. 

The mediation analysis demonstrated that fear of victimization from climate change mediated the association between exposure to climate change-related information on SNSs and the pro-environmental behaviors of users. The results are consistent with the previous findings coming from Neill and Nicholson [21] and Coelho, Pereira, Cruz, Simões, and Barata [132], who found that media significantly shape individuals’ ecological concerns, which, in turn, mediate the effect of media on the pro-environmental behaviors of people. Research shows that the existential threats [133] and emotions experienced by people mediate the effect of media exposure on their pro-environmental behaviors [134]. The results reinforced the importance of fear as a motivational factor in the context of media’s influence on individuals’ pro-environmental behaviors. When people are fearful and perceive serious threats, risks, and concerns regarding climate change, they are likely to adopt pro-environmental behaviors to avoid negative consequences [94,95]. The rapid occurrences of disasters such as floods, droughts, water scarcity, and standard food scarcity, contribute to the fear and ecological concerns of people.

The attention of people toward the media coverage and portrayals of various issues influences their perceptions and attitudes [25]. The findings showed that the attention deficit of individuals to climate change-related information on SNSs moderates the association between exposure to climate change-related information on SNSs and their fear of victimization from climate change. Similarly, the results indicated that users’ attention buffers the association between exposure to climate change-related information and pro-environmental behaviors (Figure 1 and Figure 2). The results are in line with those obtained by Reeck et al. [87] and Namkoong et al. [89], who found that the level of attention paid to information has a significant effect on the emotional stimuli and cognitive responses of users. When individuals have high dependency and attention toward the information on SNSs, their fear of victimization increases and their pro-environmental behaviors are positively and significantly influenced. Similarly, previous research suggests that SNSs play an important role in regulating the the normative perceptions and pro-environmental behaviors of individuals [8]. However, Ho, Liao, and Rosenthal [135] demonstrated that media and Internet attention significantly moderate the media’s influences on the pro-environmental and engagement behaviors of people. 

In another secondary factor, the results revealed that the decision-making self-efficacy of individuals enhances and strengthens the association between fear of victimization and pro-environmental behaviors, as shown in Figure 3. The findings are consistent with past research, suggesting that self-efficacy has a positive and significant moderating impact on individuals’ decision support systems [101], attitude dimensions, and willingness to consume functional food [136]. The effect of fear of victimization on pro-environmental behaviors is strong when individuals have great decision-making self-efficacy and self-confidence [137]. Individuals with low decision-making self-efficacy likely decrease or completely give up their efforts in difficult situations [93].

### 5.1. Theoretical Implications

This study has various significant theoretical implications to the literature on media use and environmentally-friendly behaviors. It emphasizes the mediation effect of fear of victimization on the association between the exposure to climate change-related information on SNSs and the pro-environmental behaviors of users. The study also extends EPPM by adding two new variables, namely, attention and decision-making self-efficacy. How individuals’ attention to information moderates the association between their exposure to SNSs and emotional reactions has not been examined. In this study, the significant, moderating impact of an attention deficit sheds light on the relationship between the exposure to SNSs and the socio-emotional reactions of users. Notably, the study is the first to examine the significant, moderating impact of decision-making self-efficacy on the impact of fear of victimization on people’s pro-environmental behaviors. Individuals’ self-efficacy occurs when they perceive the rewards and needs of taking actions to escape or mitigate the adverse consequences of climate change [136]. Overall, people’s emotional reactions to climate change-related information and high self-efficacy influence their pro-environmental behaviors. 

### 5.2. Practical Implications

SNSs have become important sources of information and communication. To create awareness and increase the ecological concerns of people, media organizations and governments should highlight the unfavorable outcomes of climate change and have additional environment-related advertisements. Weak fear may not attract attention, but strong fear may lead individuals to defensive strategies by blocking or ignoring climate change-related information. To encourage the engagement of people in pro-environmental behaviors, media and other information sources should report the environmental issues in a factual, less personal manner without exaggerated adverse impacts. 

When users encounter a large amount of information on SNSs, they place too much emphasis on irrelevant information and lose attention when it comes to important parts. Similarly, users’ trust or perceived risk of inaccurate content or information overload can affect their engagement in and attention toward online information. To emphasize the importance of climate change and attract the attention of users toward it, government organizations and policy makers must consider users’ experience and their trust as crucial aspects of their attention toward online pro-environmental behaviors. Moreover, government organizations and policy makers should enhance information quality and system quality to attract users’ attention toward and improve their trust in information via SNSs. Governmental and non-governmental organizations and authorities concerned must also run their campaigning programs and messages related to climate change on different channels to further emphasize the importance of issues in the public mind and direct their attention toward information on SNSs. 

Climate change has adverse impacts and is a threat to future generations. However, the abilities, attention, self-confidence, and effectiveness of individuals have impacts on their behaviors. Individual-based actions have limited impacts until and unless a collective effort for mitigation is initiated or the threats of environmental issues are avoided [78]. In collectivist cultures, people have unity; they integrate and perceive themselves as confident to address the confronted challenges. To gain institution-based trust, encourage and enhance the decision-making self-efficacy of individuals, and motivate and engage them toward pro-environmental behaviors, media organizations and government policy makers should propose effective fear appeal strategies in light of different cultural backgrounds and personality traits. 

### 5.3. Limitations and Future Directions

This research has various limitations that may be considered in future studies. First, the generalizability of the study for the whole China is limited because the data were collected from a single large university. Future studies could consider multiple samples from different age groups, different sectors, and different countries to increase the generalizability of this study. Second, the research is based on a cross-sectional design, which does not contribute to causal inferences. Future researchers must conduct longitudinal or experimental studies to test the causal relationships of the constructs. Third, the study argues that the impact of exposure to SNSs on pro-environmental behaviors may be related to users’ fear of victimization from environmental issues. However, different media sources may describe environmental issues in different ways. Future researchers should examine the emotional reactions of audiences toward separate media types (television, radio, and newspapers) to determine clearer pictures of media use and pro-environmental behaviors. SNSs provide individuals with convenient outlets for acquiring awareness of environmental issues. However, due to their fast-forwarding functionality, SNSs facilitate the dissemination of misinformation, which eventually contributes to negative effects on users [138,139,140]. Therefore, in future, scholars need to consider the impacts of perceived inaccurate/disinformation on people’s fear of victimization and pro-environmental behaviors. Last, this study examined SNS users’ pro-environmental behaviors in general. Its scope can be extended by considering some specific pro-environmental behaviors. 

## 6. Conclusions

In summary, this research offers a significant contribution to SNS and behavioral studies. By employing a quantitative survey approach and using cross-sectional data, the study found that exposure to climate change-related information via SNSs elicits fear of victimization from climate change in users and positively influences their pro-environmental behaviors. The attention of individuals toward climate change-related information on SNSs also significantly moderates the association of exposure to climate change-related information on SNSs with fear of victimization and pro-environmental behaviors. Similarly, decision-making self-efficacy significantly moderates the relationship between fear of victimization from climate change and pro-environmental behaviors. The findings illustrate the importance of SNSs in educating and shaping the perceptions of people about climate change. With this study, promoting the pro-environmental behaviors of people by discussing and sharing various types of information related to climate change on SNSs may be made easy for concerned governments and non-government institutions. Moreover, policymakers must understand whether and to what extent cognitive factors can influence individuals’ pro-environmental behaviors to effectively design public policies. This study is noticeably the first stride in investigating the relationship between exposure to climate change-related information via SNSs and pro-environmental behaviors, with the belief that the current results can encourage future studies to continue this promising line of investigation.

## Figures and Tables

**Table 1:** Demographics of respondents.

Variables	Classes	Frequency (%)
Gender	Male	217 (53.4)
	Female	189 (46.6)
Age	18–21 years	128 (31.5)
	22–25 years	161 (39.6)
	26–30 or above years	117 (28.8)
Education	Bachelor	123 (30.3)
	Master	149 (36.7)
	Ph.D.	134 (33.0)

**Table 2:** Exploratory factor analysis.

Constructs	Items	FL	CA	AVE	CR
	ESNS1	0.868			
Exposure to SNSs	ESNS2	0.843	0.939	0.736	0.893
	ESNS3	0.863			
	Fear1	0.708			
Fear of victimization	Fear2	0.839			
	Fear3	0.737	0.867	0.616	0.865
	Fear4	0.847			
	PEB1	0.836			
	PEB2	0.806			
	PEB3	0.845			
Pro-environmental behaviors	PEB4	0.904	0.964	0.749	0.964
	PEB5	0.921			
	PEB6	0.820			
	PEB7	0.865			
	PEB8	0.898			
	AD1	0.886			
	AD2	0.870			
	AD3	0.899			
Attention deficit	AD4	0.899	0.973	0.805	0.971
	AD5	0.936			
	AD6	0.895			
	AD7	0.903			
	AD8	0.889			
	DSE1	0.820			
	DSE2	0.780			
	DSE3	0.886			
Decision-making self-efficacy	DSE4	0.886	0.955	0.739	0.944
	DSE5	0.900			
	DSE6	0.880			

Note: FL = factor loadings, CA = Cronbach’s alpha, CR = composite reliability, AVE = average variance extracted, ESNS = exposure to climate change-related information on SNSs, Fear = fear of victimization, PEB = pro-environmental behaviors, AD = attention deficit, DSE = decision-making self-efficacy.

**Table 3:** Bivariate correlations.

	Mean	SD	1	2	3	4	5	6	7	8
1.Gender	1.15	0.360	1							
2.Age	2.07	0.843	−0.102 *	1						
3.Education	1.99	10.08	0.275 **	0.235 **	1					
4.ESNS	3.86	10.03	0.024	0.026	0.040	(**0.85**)				
5.FEAR	4.17	0.75	0.054	−0.004	−0.035	0.411 **	(**0.78**)			
6.PEB	4.11	0.62	−0.012	−0.016	0.000	0.246 **	0.403 **	(**0.86**)		
7.AD	2.16	0.88	0.072	0.028	0.040	−0.491 **	−0.271 **	−0.186 **	(**0.90**)	
8.DSE	4.14	0.76	−0.063	0.022	−0.074	0.324 **	0.411 **	0.465 **	−0.170 **	(**0.86**)

** Correlation is significant at the 0.01 level (2-tailed). * Correlation is significant at the 0.05 level (2-tailed). Note: ESNS = exposure to climate change-related information on SNSs, Fear = fear of victimization, PEB = pro-environmental behaviors, AD = attention deficit, DSE = decision-making self-efficacy. The bold values in parentheses represent discriminant validity.

**Table 4:** Mediation testing.

Path (Direct Path)	Estimates (β)		SE	
ESNSs → Fear	0.299 **		0.033	
ESNSs → PEB	0.149 **		0.029	
Fear → PEB	0.301 **		0.041	
Indirect Effect				
Path (Indirect Path)	Effect	SE	LL 95% CI	UL 95% CI
ESNSs → Fear → PEB	0.090	0.016	0.094	0.202

** *p* < 0.01, Note: ESNSs = exposure to climate change-related information on SNSs, Fear = fear of victimization, PEB = pro-environmental behaviors, LL = lower limit confidence interval, UL = upper limit confidence interval, SE = standard error.

**Table 5:** Moderation analysis.

Predictors	Outcomes			
	Fear of Victimization		Pro-Environmental Behaviors	
	β	t	β	t
Exposure to SNSs	0.282	7.074 **	0.139	3.021
Attention	−0.094	−2.301 *	−0.076	−2.004
ESNSs × Attention	−0.090	−2.689 **		
ESNSs × Attention			−0.090	−2.197 *
Fear of victimization			0.265	5.442 **
DSE			0.305	5.424 **
Fear of victimization × DSE			0.267	5.720 **

* *p* < 0.05; ** *p* < 0.01; Note: ESNSs = exposure to climate change-related information on SNSs, DSE = decision-making self-efficacy.

## Data Availability

The data will be made available from the authors upon request.
